# Omega-3 fatty acids enhance the beneficial effect of BCAA supplementation on muscle function following eccentric contractions

**DOI:** 10.1080/15502783.2022.2117994

**Published:** 2022-09-08

**Authors:** Yosuke Tsuchiya, Kenichi Yanagimoto, Norihiko Sunagawa, Hisashi Ueda, Katsunori Tsuji, Eisuke Ochi

**Affiliations:** a Meiji Gakuin University, Center for Liberal Arts, Laboratory of Health and Sports Sciences, Kanagawa, Japan; b Food Function R&D Center Nippon Suisan Kaisha Ltd, Tokyo, Japan; c Teikyo Heisei University, Faculty of Modern Life, Tokyo, Japan; d Teikyo Heisei University, Faculty of Health and Medical Science, Chiba, Japan; e Hosei University, Faculty of Bioscience and Applied Chemistry, Tokyo, Japan; f Hosei University, Graduate School of Sports and Health Studies, Tokyo, Japan

**Keywords:** Ergogenic aid, supplement, sports nutrition, essential amino acids, omega-3 fatty acids

## Abstract

**Background:**

This study investigated the combined effect of branched-chain amino acids (BCAA) and fish oil (FO) on muscle damage caused by eccentric contractions (ECCs) of the elbow flexors, with a special focus on muscular function.

**Methods:**

Twenty-nine untrained male participants were enrolled in this double-blind, placebo-controlled, parallel study. The participants were randomly assigned to the placebo (PL) group (n = 9), BCAA supplement group (n = 10), and BCAA+FO supplement group (n = 10). The BCAA+FO group consumed eicosapentaenoic acid (EPA) 600 mg and docosahexaenoic acid (DHA) 260 mg per day for 8 weeks, while the BCAA and BCAA+FO groups consumed 9.6 g per day for 3 days prior to and until 5 days after ECCs. Participants performed six sets of 10 ECCs at 100% maximal voluntary contraction (MVC) using dumbbells. Changes in MVC torque, range of motion (ROM), muscle soreness using visual analog scales, upper circumference, muscle thickness, echo intensity, and serum creatine kinase (CK) were assessed before, immediately after, and 1, 2, 3, and 5 days after ECCs.

**Results:**

The MVC torque was significantly higher in the BCAA+FO group than in the PL group immediately after ECCs (p < 0.05) but not in the BCAA group. Both BCAA and BCAA+FO groups showed greater ROM and lower muscle soreness than the PL group (p < 0.05). CK was significantly lower in the BCAA group than in the PL group at 5 days after ECCs (p < 0.05).

**Conclusions:**

This study reveals that supplementation with BCAA and FO may favorably impact immediate recovery of peak torque production. Alternatively, in comparison to PL group, BCAA supplementation favorably reduces creatine kinase.

## 1. Introduction

Exercise involving exhaustive eccentric contractions (ECCs) can cause muscle soreness and muscle damage, including reduced maximal strength, limited range of motion (ROM), muscle swelling, and increased levels of creatine kinase (CK) [1]. Consequently, ECCs can lead to discomfort and decreased performance. Therefore, it is important to minimize decreases in muscle function and muscle soreness as well as promote the repair of muscle damage after exercise.

Branched-chain amino acids (BCAAs) such as valine, leucine, and isoleucine are among the nine essential amino acids for humans, and account for 35% of the essential amino acids in muscle proteins and 40% of the preformed amino acids required by mammals [2]. BCAAs have several nutritional and functional roles, including serving as an energy source during exercise [3], acting as a signaling factor of protein synthesis [4], and facilitating anti-inflammatory and immune responses [5]. In particular, 9.6 g of BCAA supplementation taken three days before performing elbow flexor ECCs was effective at attenuating muscle soreness, limiting losses of ROM, increased upper arm circumference, and increased serum CK [6]. However, Foure et al. [7] reported that 7 g of BCAA per day had no effect on the recovery of muscle damage after maximal voluntary isometric contraction of the quadriceps in untrained males. In contrast, BCAA supplementation enhanced the recovery of muscle strength following ECCs in men who had undergone resistance training [8].

Previous studies have revealed that fish oil (FO) including eicosapentaenoic acid (EPA) and docosahexaenoic acid (DHA) positively affected the symptoms of muscle damage [9,10]. Our previous research found that 8-week FO supplementation attenuates reductions in muscle strength and ROM and increases in muscle soreness, CK, and muscle stiffness following exercise involving ECCs [11,12]. As for the mechanisms of the observed effects, it has been suggested that FO is incorporated into phospholipids, which are a major component of the cell membrane, and thereby inhibit the effects of inflammation and reactive oxygen species [13].

Although the detailed mechanisms are not known, several previous studies focused on the effect of combined supplementation on muscle damage [14–17]. Specifically, supplements containing BCAA and vitamin B (vitamin B12, B6, B9, and B5) [14], glucose [15], and BCAA and taurine [6] have shown combined effects on muscle damage. Indeed, one review article recommended that the combined effect of BCAA and other nutrients should be examined further [18], while another suggested that the combined effects of FO and other supplements be investigated [19]. However, no study has yet investigated the combined effects of BCAA and FO on muscle damage after performing acute ECCs.

Therefore, the purpose of this study was to investigate whether FO supplementation enhances the individual effect of a single dose of BCAA supplementation on muscle damage after performing ECCs. We hypothesized that combined BCAA and FO supplementation would have greater beneficial effects on the muscle damage after ECCs compared with supplementation of either one alone.

## 2. Materials and methods

### Participants

A total of 29 healthy men (age, 19.5 ± 1.1 years; height, 170.5 ± 6.1 cm; weight, 66.2 ± 9.5 kg; body mass index (BMI), 22.7 ± 2.5 kg/m^2^) were recruited for this study. The participants reported having no food allergies. In addition, they did not perform in any regular resistance training for at least one year prior to participation in this study. They were requested to avoid participating in other clinical trials and interventions, including those involving hot baths, cold baths, massage, stretching, strenuous exercise, excessive consumption of food or alcohol, and to refrain from taking any supplements or medication during the experimental period. All participants were provided with detailed explanations of the study protocol prior to participation and signed an informed consent form in accordance with the Declaration of Helsinki before being enrolled in this study. This study was approved by the Ethics Committee for Human Experiments at Teikyo Heisei University (ID: R02-34). Moreover, the study was registered at the University Hospital Medical Information Network Clinical Trials Registry (UMIN-CTR identifier: UMIN000042925).

### Study design

The study employed a double-blind, placebo-controlled, parallel-group trial design. The participants were randomly assigned to one of the three groups using a table of random numbers to minimize the intergroup differences in prior activity, age, height, body weight, and BMI. The placebo (PL) group and the BCAA group consumed a placebo pill daily, whereas the BCAA+ FO group consumed an EPA-rich FO for 8 weeks before the exercise experiment and for five days after exercise was completed. In addition, the BCAA group and the BCAA+ FO group consumed BCAA for three days before the exercise experiment. On the day of the exercise test, muscle damage markers were assessed in the non-dominant arm before exercise. Immediately after these baseline measurements, the participants performed ECCs with the same arm. The participants were instructed to eat a light meal 2 or more hours before arriving at the laboratory. All measurements were performed immediately before, immediately after, and 1, 2, 3, and 5 days after exercise.

### Supplements

All groups consumed eight soft-gel capsules per day for 8 weeks before (not after) the exercise experiment and then up to 5 days after. The BCAA+FO group consumed eight 300-mg EPA-rich FO softgel capsules (600 mg EPA, 260 mg DHA; Nippon Suisan Kaisha Ltd., Tokyo, Japan) per day, following the procedures in previous studies [12]. The intake of supplements in the BCAA group and the BCAA+FO group was changed to 3 times a day from 3 days before the exercise experiment. The daily intake of 9.6 g BCAA (Nippon Suisan Kaisha Ltd., Tokyo, Japan) was based on previous studies [6]. On the day of the exercise experiment, the participants ingested 9.6 g of placebo or BCAA immediately before the exercise (not after). The placebo supplement was compounded to the same volume, color, and taste as the BCAA by using similar proportions of cornstarch for the double-blind method.

### Nutrition survey

The nutritional status of each participant was assessed before and after the 8-week supplementations by using a food frequency questionnaire based on food groups, as described in previous studies (FFQg, version 5, Kenpakusha, Tokyo, Japan) [11].

### Eccentric contractions (ECCs)

To perform ECCs, participants sat on a preacher curl bench with their shoulder joint angled at 45° flexion. Each participant used a dumbbell having a weight equal to 100% of their maximal voluntary isometric contraction MVC at an elbow joint angle of 90°. Based on previous studies [11,20], the ECCs comprised six sets of 10 maximal voluntary ECCs of the elbow flexors with a resting period of 90s between each set. The participants were handed the dumbbell with their elbow in the flexed position (90°) and were instructed to lower it to a completely extended position (0°) at an approximately constant speed (30°/s) in time (3 s), using a metronome. Then, the investigator took the dumbbell from the participant, and the participant returned their arm to the starting position for the next ECC.

### Maximal voluntary isometric contraction (MVC) torque

Each participant performed two 5-s MVCs at an elbow joint angle of 90° with a 60-s rest between the contractions. The peak torque at each angle was considered to be the MVC torque. The torque signal was amplified using a strain amplifier (LUR-A-100NSA1; Kyowa Electronic Instruments, Tokyo, Japan). The analog torque signal was converted to a digital signal by a 16-bit analog-to-digital converter (Power-Lab 16SP; AD Instruments, Bella Vista, Australia). The sampling frequency was set to 10 kHz and the measurement was performed as previously described [21]. The test–retest reliability of the MVC measurements based on the coefficient of variation (CV) was 1.7%.

### Range of motion of the elbow joint

To examine the elbow joint ROM, two elbow joint angles (extended and flexed) were measured using a goniometer (Takase Medical, Tokyo, Japan). The extended joint angle was recorded while the participant attempted to completely extend the joint with the elbow held by his side and the hand in supination [22]. The flexed joint angle was determined, while the participant attempted to completely flex the joint from an equally and completely extended position with the hand in supination. The ROM was calculated by subtracting the flexed joint angle from the extended joint angle. The test–retest reliability of the ROM measurements based on the CV was 0.8%.

### Muscle soreness using visual analogue scales

Muscle soreness in the elbow flexors was assessed using a 100-mm visual analog scale (VAS) ranging from 0 (‘no pain’) to 100 (“the worst pain imaginable”), in line with previous studies [10,22]. The participants attained a relaxed state with the arm in a natural position; the investigator then palpated the upper arm using a thumb, and the participant indicated his pain level on the visual scale. All tests were performed by the same investigator who was trained to apply the same pressure over time and across participants. The test–retest reliability of the VAS measures based on the CV was 2.2%.

### Blood sample

The participants fasted for 8 h before a trained doctor obtained blood samples from their forearms. The blood samples were allowed to clot at room temperature (25°C) and were then centrifuged at 3,000 rpm for 10 min at 4°C. The serum was extracted and stored at −20°C until analysis. The serum levels of dihomo-gamma-linolenic acid (DGLA), arachidonic acid (AA), EPA, and DHA were measured. In addition, we evaluated serum creatine kinase (CK) as a marker of muscle damage.

### Upper arm circumference

The upper arm circumference was measured 9 cm above the elbow joint using a tape measure while the participants were standing with their arms relaxed by their side, as in a previous study [11]. Measurement marks were maintained during the experimental period using a semi-permanent marker, and the measurements were taken by the same experienced investigator. The mean value of three measurements was used for further analysis. The test–retest reliability of the measurements based on the CV was 2.1%.

### Muscle thickness and echo intensity

B-mode ultrasound pictures of the upper arm were obtained using the biceps brachii via ultrasound (SONIMAGE HS1; Konika Minolta, Tokyo, Japan), and the probe was placed 9 cm from the elbow joint at the position marked for the measurement of the upper arm circumference. The same gains and contrast were used over the experimental period. The transverse images were transferred to a computer as bitmap files (.bmp) and analyzed using a computer. Scanned images of each muscle were transferred to a personal computer and the thickness of biceps brachii was manually calculated via tracing using computer image analysis software (ImageJ; National Institutes of Health, Bethesda, MD). The average muscle echo intensity of the region of interest (20 × 20 mm) was calculated using the same software to provide a grayscale histogram (0, black; 100, white) for the region, as described in a previous study [11]. The test–retest reliability of the muscle echo intensity and thickness measurements based on CV were 2.1% and 1.4%, respectively.

### Statistical analyses

All analyses were performed using the SPSS ver 26.0 (IBM Corp., Armonk, NY). Values are expressed as means ± standard deviation (SD). MVC torque, ROM, circumference, muscle thickness, and echo intensity of values before exercise to 5 days after exercise were calculated based on relative changes from the baseline. MVC, ROM, muscle soreness, circumference, muscle thickness, echo intensity, and blood data of the PL group and BCAA group or the PL group and BCAA+FO group were compared using a two-way repeated-measure analysis of variance (ANOVA). When a significant primary effect or interaction was found, Bonferroni’s correction was performed for post-hoc testing. A p-value of < 0.05 was considered statistically significant.

## 3. Results

### Participant characteristics and food frequency surveys

No significant differences were observed among the PL group, the BCAA group, and the BCAA+FO group in terms of age, height, body weight, and BMI (PL group, n = 9; age, 19.6 ± 1.1 years; height, 169.8 ± 6.1 cm; weight, 64.7 ± 6.5 kg; body fat, 17.1 ± 2.6%; and BMI, 22.4 ± 2.0; BCAA group, n = 10; age, 19.8 ± 1.3 years; height, 170.3 ± 6.5 cm; weight, 67.9 ± 13.2 kg; body fat, 16.0 ± 4.7%; and BMI, 23.3 ± 3.4, BCAA+FO group, n = 10; age, 19.2 ± 0.6 years; height, 171.3 ± 6.1 cm; weight, 65.7 ± 8.1 kg; body fat, 16.0 ± 2.1%; and BMI, 22.4 ± 1.9). The results of the food frequency questionnaire showed no difference in nutrition status among the PL group, the BCAA group, and the BCAA+FO group before and after the experiment (Table 1).

**Table 1.** Changes of food frequency surveys and serum dihomo-gamma-linolenic acid, arachidonic acid, eicosapentaenoic acid, docosahexaenoic acid.

Food frequency surveys		PL (n = 9)	BCAA (n = 10)	BCAA+FO (n = 10)
Energy (kcal)	Before supplementations	2023.6 ± 670.0	2396.3 ± 426.1	2459.0 ± 1043.3
	After supplementations	1836.9 ± 665.0	2357.3 ± 1260.0	2408.3 ± 983.3
Protein (g)	Before supplementations	71.2 ± 35.5	93.1 ± 18.9	86.3 ± 37.9
	After supplementations	73.5 ± 40.4	92.7 ± 69.6	90.4 ± 43.1
Fat (g)	Before supplementations	67.7 ± 23.4	91.9 ± 20.8	80.0 ± 46.6
	After supplementations	70.1 ± 20.7	81.4 ± 44.6	84.8 ± 42.1
Carbohydrate (g)	Before supplementations	274.4 ± 79.1	290.1 ± 87.0	336.4 ± 131.5
	After supplementations	220.0 ± 87.6	302.3 ± 143.1	311.0 ± 133.0
Omega-3 fatty acids (g)	Before supplementations	2.2 ± 1.2	2.8 ± 0.8	2.6 ± 1.6
	After supplementations	2.7 ± 1.8	3.1 ± 1.8	2.8 ± 1.3
Serum fatty acid				
Dihomo-gamma-linolenic acid (μg/ml)	Before supplementations	188.7 ± 42.0	170.8 ± 45.1	171.2 ± 21.3
	After supplementations	178.1 ± 24.2	157.2 ± 43.9	166.5 ± 24.5
Arachidonic acid (μg/ml)	Before supplementations	32.0 ± 12.3	37.0 ± 7.0	32.6 ± 10.3
	After supplementations	27.8 ± 9.8	31.7 ± 7.7	29.0 ± 8.7
Eicosapentaenoic acid (μg/ml)	Before supplementations	29.3 ± 15.4	22.3 ± 11.1	33.2 ± 25.3
	After supplementations	24.2 ± 21.3	17.8 ± 13.3	60.9 ± 16.5†*
Docosahexaenoic acid (μg/ml)	Before supplementations	68.1 ± 17.9	71.6 ± 24.9	72.7 ± 25.2
	After supplementations	64.2 ± 14.9	60.5 ± 21.1	82.2 ± 16.2*

† p < 0.05 for the difference from the before supplementations value. * p < 0.05 for the difference between BCAA+FO group and PL, BCAA group.

### Changes in serum DGLA, AA, EPA, and DHA before and after the 8-week supplementation

The changes in serum fatty acids are shown in Table 1. No significant changes in DGLA, AA, EPA, and DHA levels were observed in the PL group and the BCAA group before and after supplementation. However, the EPA level after supplementation in the BCAA+FO group was significantly higher than before supplementation (p < 0.05). EPA and DHA levels were significantly higher in the BCAA+FO group than in the PL group and the BCAA group after the 8-week supplementation (p < 0.05).

### Maximal voluntary isometric contraction torque

**Figure 1.**
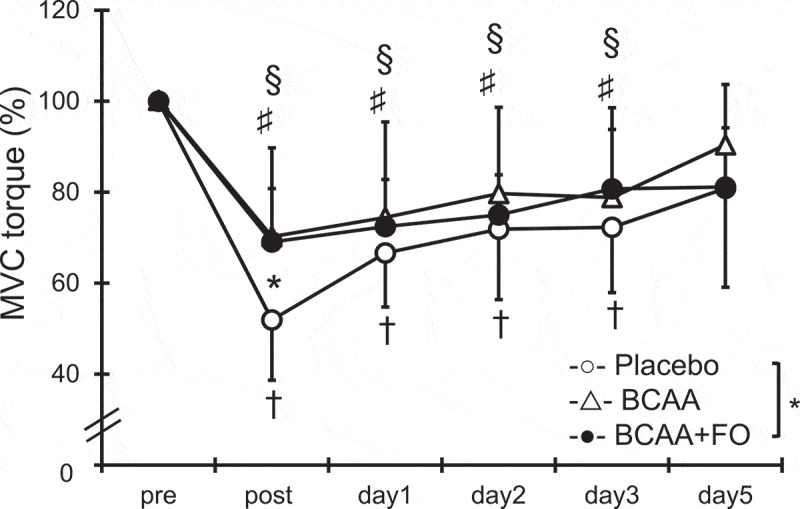
Changes (mean ± SD) in maximal voluntary isometric contraction (MVC) torque at 90° measured before (pre) and immediately after (post) the eccentric contractions exercise and 1, 2, 3, and 5 days after in the placebo (PL) group, the BCAA group, and the BCAA+FO group. * p < 0.05 for the difference between the PL group and the BCAA+FO group. † p < 0.05 for the difference from the pre-exercise value in the PL group. § p < 0.05 for the difference from the pre-exercise value in the BCAA group. ♯ p < 0.05 for the difference from the pre-exercise value in the BCAA+FO group.

A significant interaction effect was found between the PL group and the BCAA+FO group (Figure 1, p < 0.05). Compared with the pre-exercise value, the MVC torque at 90° elbow angle in the three groups significantly decreased immediately after exercise and did not return to baseline until day 3 after exercise (p < 0.05; Figure 1). There was a significant difference between the BCAA+FO group (69.1 ± 11.7%) and the PL group (51.9 ± 13.3%) immediately after exercise (p < 0.05). In contrast, no significant difference was found between the PL group and the BCAA group.

In the pdf file, the fig 1 looks too big, please rotate 90degs and insert to the text that works well.

### Range of motion of the elbow joint

**Figure 2.**
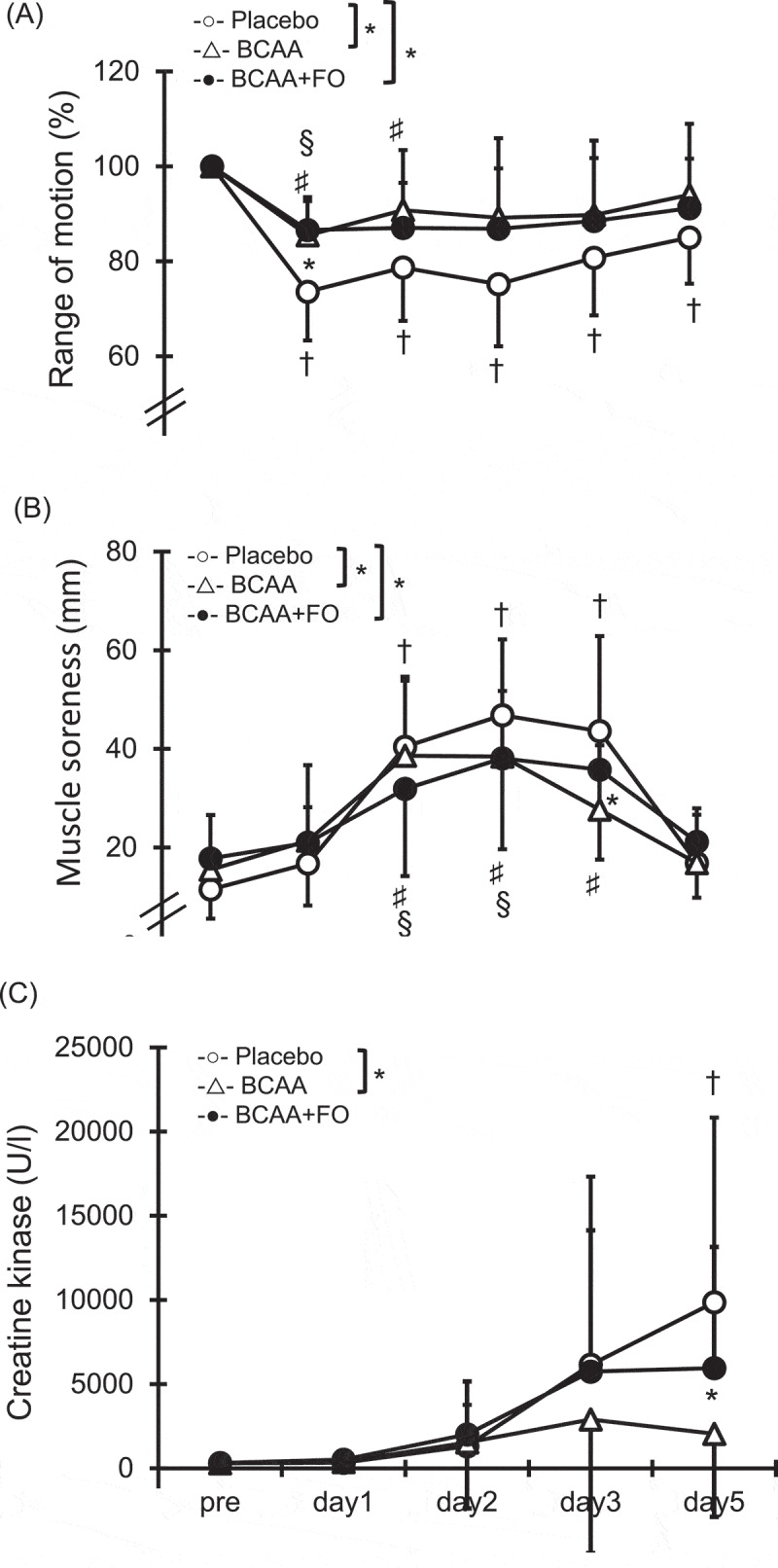
Changes (mean ± SD) in range of motion (ROM) (A) and muscle soreness (B), and blood serum creatine kinase level (C) measured before (pre) and immediately after (post) the eccentric contractions exercise and 1, 2, 3 and 5 days after in the placebo (PL) group, the BCAA group, and the BCAA+FO group. * p < 0.05 for the difference between PL and BCAA group. † p < 0.05 for the difference from the pre-exercise value in the PL group. § p < 0.05 for the difference from the pre-exercise value in the BCAA group. ♯ p < 0.05 for the difference from the pre-exercise value in the BCAA+FO group.

A significant interaction effect was found between the PL group and the BCAA group and between the PL group and the BCAA+FO group (Figure 2A, p < 0.05). Compared with the pre-exercise value, ROM had significantly decreased immediately after exercise and remained until 5 days after exercise in the PL group. In the BCAA group, ROM was significantly decreased only immediately after exercise, but returned to baseline 1 day after exercise. Moreover, the BCAA+FO group was significantly decreased immediately after and 1 day after exercise, but returned to baseline 2 days after exercise. In addition, ROM was significantly higher in the BCAA group than in the PL group immediately after exercise (BCAA group, 85.5 ± 8.0%; PL group, 73.6 ± 10.3%; p < 0.05).

### Muscle soreness using visual analogue scale

A significant interaction effect was found between the PL group and the BCAA group and between the PL group and the BCAA+FO group (Figure 2B, p < 0.05). In the PL group and the BCAA+FO group, a greater muscle soreness developed at 1, 2, and 3 days after exercise compared with the pre-exercise values. In the BCAA group, a greater muscle soreness developed at 1 and 2 days after exercise compared with the pre-exercise values. There was a significant difference in muscle soreness between the PL group (43.6 ± 19.3 mm) and the BCAA group (27.7 ± 13.0 mm) at 3 days after exercise (p < 0.05).

### Blood serum creatine kinase (CK) analysis

A significant interaction effect was found between the PL group and the BCAA group (Figure 2C, p < 0.05). In the PL group, the serum CK level at 5 days after exercise was higher than the pre-exercise values (p < 0.05). In contrast, no significant differences were found in the BCAA group and the BCAA+FO group. There was a significant difference in serum CK level between the BCAA group (2051.1 ± 4940.2 U/l) and the PL group (9862.6 ± 10,971.1 U/l) at 5 days after exercise (p < 0.05).

### Upper arm circumference, muscle thickness, and echo intensity

**Figure 3.**
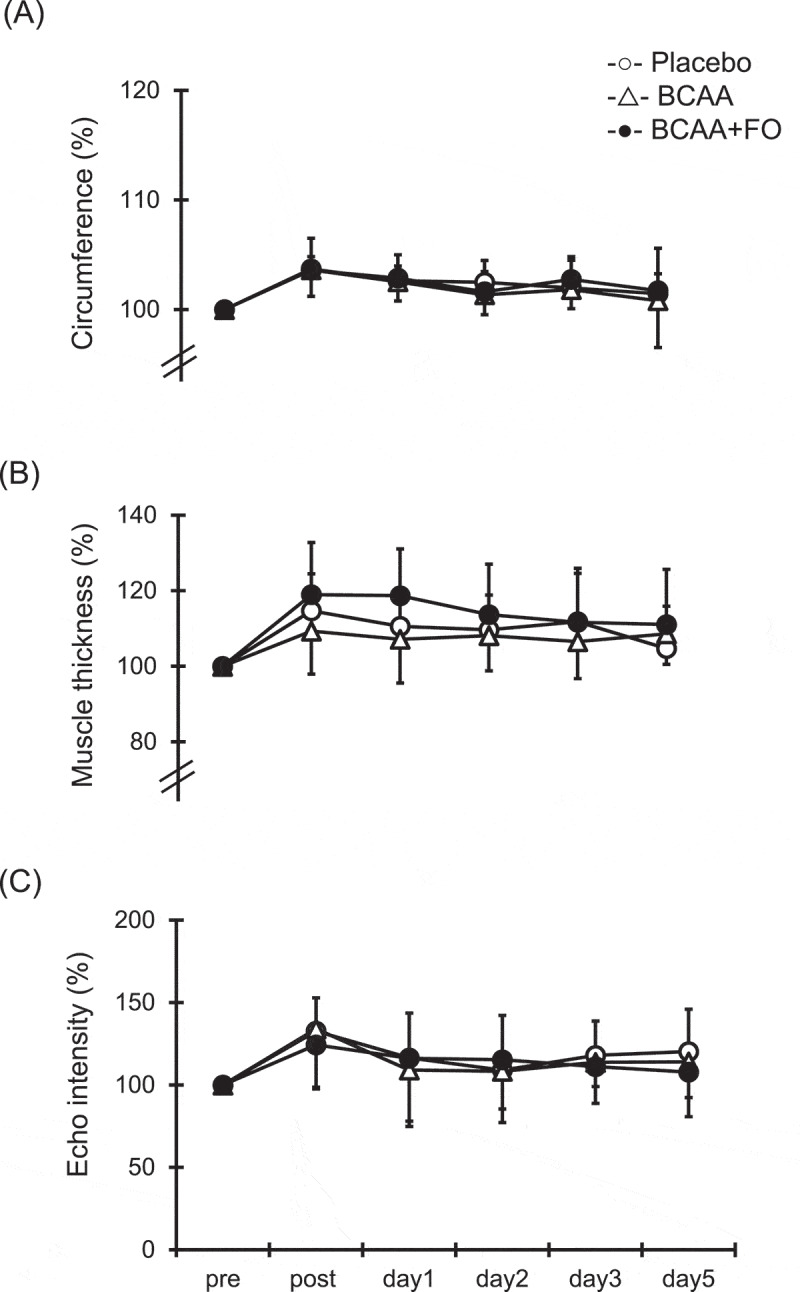
Changes (mean ± SD) of circumference (A), muscle thickness (B) and echo intensity (C), measured before (pre) and immediately after (post) the eccentric contractions exercise and 1, 2, 3 and 5 days after in the placebo (PL) group, the BCAA group, and the BCAA+FO group.

No significant differences in upper arm circumference were observed at any point in the three groups (Figure 3A). Also, no significant differences in muscle thickness and muscle echo intensity were observed at any point in the three groups (Figure 3B,C).

## 4. Discussion

This study investigated the efficacy of BCAA and BCAA+FO supplementation on their ability to mitigate muscle damage after performing ECCs. We confirmed that the combination of BCAA and FO attenuated the decrease in muscle strength immediately after performing ECCs. The present study also reported that BCAA and BCAA+FO improved ROM and reduced soreness after completing damaging exercise and BCAA supplementation reduced CK values five days after eccentric exercise.

The MVC in the BCAA+FO group was significantly higher than that in the PL group after performing ECCs, but not in the BCAA group. It has been shown that 20 g/day BCAA supplementation administered 7 days before and 4 days after performing ECCs of knee extensors (with an additional 20 g immediately before and after the exercise) attenuated reductions in MVC and led to earlier recovery in participants who had undergone resistance training [23]. However, Foure et al. [7] reported that 7.0 g of BCAA per day had no effect on the recovery of MVC of knee extensors after performing ECCs in men who had not undergone resistance training. Likewise, in a study by Jackman et al. [24], no differences were demonstrated in MVC in men who had not undergone resistance training with 29.3 g BCAA supplementation per day. Therefore, single intake of BCAA may not prevent muscle dysfunction after performing ECCs in participants who have not undergone resistance training. In contrast, several studies have revealed that FO supplementation attenuated reduced muscle strength after performing ECCs of elbow flexors [10–12]. We found that intake of FO inhibits the reduction in muscle strength [12]. The mechanism of temporal strength loss after performing ECCs is assumed to be due to excitation–contraction coupling failure [25]. It has been demonstrated that FO supplementation attenuates reduced muscle strength after performing ECCs concomitant with protection against motor nerve function [11]. Although single intake of BCAA tended to attenuate reduced muscle strength, no statistically significant effect was observed in this study. Considering these factors, BCAA alone does not appear to be sufficient for preventing reduced muscle strength after performing ECCs, and thus the results suggest that FO plays an important role in the protection of muscle function.

This study reveals that BCAA supplementation improves ROM immediately after exercise and improved soreness three days after exercise. Ra et al. [6] reported that the intake of BCAA for 3 days before performing ECCs (9.6 g/day) attenuated the limited ROM and muscle soreness. In addition, Rahimi et al. [18] reviewed randomized clinical trials and concluded that BCAA supplementation, both before and during recovery days after exercise, is able to attenuate muscle soreness. Although the mechanism underlying muscle soreness after performing ECCs is not well understood, it seems likely to be related to inflammation, particularly to the connective tissue elements [26] that sensitize nociceptors in muscle [27]. In addition, limited ROM after ECCs has also been attributed to an inflammatory response in the myofibrils, which leads to an increase in passive stiffness [28]. Nicastro et al. [29] suggested that BCAA supplementation decreases the inflammatory response in damaged muscle by increasing the availability of amino acids as substrates for immune cells. Regarding FO, a recent double-blind, placebo-controlled, parallel study showed that daily FO supplementation for 8 weeks of (EPA 600 mg, DHA 260 mg) led to higher attenuation of limited ROM and muscle soreness compared with a placebo [12]. Because FO has also anti-inflammatory effects that reduce levels of interleukin-6 [10,30], the attenuation of limited ROM and muscle soreness might be attributed to these anti-inflammatory effects. Of note, no differences were found in ROM and muscle soreness between the single BCAA and combined supplementation groups, and no combined effects of BCAA and FO were observed in either parameter.

Although there was a significant difference in CK between the BCAA group and the PL group, no such difference was found between the BCAA+FO group and the PL group. Many studies have reported that BCAA supplementation attenuated increased serum CK after performing ECCs [7,16,23,31]. A systematic review and meta-analysis concluded that BCAA significantly reduces efflux of CK after exercise [18]. In contrast, the effects of FO supplementation on increased CK activity have been controversial. Tartibian et al. [30] reported that intake of FO for 30 days reduced elevation of CK after performing ECCs from 40 min after performing the bench stepping exercise. Similarly, FO supplementation for 4 weeks alleviated the increase in CK after performing ECCs of elbow flexions [32]. In contrast, previous studies have found no differences in serum CK between FO supplementation and placebo conditions [10,33]. CK is more indicative of sarcolemma disruption and accordingly causes the cytosolic enzymes to exude from the cell into the blood [18,27]. Rahimi et al. [18] speculated that muscle membrane integrity might be maintained to a greater extent by BCAA supplementation. However, the mechanism by which BCAA assists in repairing/preserving the muscle sarcolemma membrane has yet to be elucidated. Future research should examine the relationship between amino acid availability and sarcolemma remodeling. Moreover, CK level was lower in the BCAA+FO group than in the BCAA-only group in this study. Therefore, it is necessary to clarify the offsetting effects of combining intake of BCAA with other supplements.

Neither intake of BCAA alone nor combined intake of BCAA and FO had any effect on swelling and echo intensity. Previous studies have reported that BCAA supplementation attenuated the increase in circumference [6]. Similarly, FO supplementation for 8 weeks inhibited increases in circumference [12]. However, several previous studies have reported that the consumption of EPA and DHA supplementation did not attenuate muscle swelling after performing ECCs [10,11,34]. Therefore, there is presently no consensus on muscle swelling measured using a tape measure. Increased echo intensity reflects the amount of free water or edema [35]. To our knowledge, no studies have examined the effect of BCAA supplementation on echo intensity. Above all, more studies are needed to clarify the effect on echo intensity after performing ECCs.

## 5. Conclusions

In conclusion, the results of this study showed that combining FO and BCAA supplementation has a beneficial effect in terms of immediately attenuating loss of strength after performing eccentric contractions in untrained males. Therefore, combined supplementation with BCAA and FO may be a useful strategy for attenuating muscle dysfunction. This study showed that the BCAA supplementation had no statistical effect on muscle function despite improved CK, ROM, and muscle soreness. Further study is needed to examine the relationship between attenuated the limited ROM, increased CK, and muscle soreness and muscle function in more details.

## Data Availability

The datasets used and analyzed during the current study are available from the corresponding author on reasonable request.

## Abbreviations

AA: Arachidonic acid; ANOVA: Analysis of variance; BCAA: Branched-chain amino acid; CK: Creatine kinase; CV: Coefficient of variation; DGLA: Dihomo-gamma-linolenic acid; DHA: Docosahexaenoic acid; ECCs: Eccentric contractions; EPA: Eicosapentaenoic acid; FO: Fish oil; MVC: Maximal voluntary contraction; PL: Placebo; ROM: Range of motion; SD: Standard deviation

## References

[1] Clarkson PM, Sayers SP. Etiology of exercise-induced muscle damage. Can J Appl Physiol = Revue Canadienne de Physiologie Appliquee. 1999;24:234–248. 10364418 10.1139/h99-020

[2] Shimomura Y, Murakami T, Nakai N, et al. Exercise promotes bcaa catabolism: effects of bcaa supplementation on skeletal muscle during exercise. J Nutr. 2004;134:1583s–1587s. 15173434 10.1093/jn/134.6.1583S

[3] Platell C, Kong SE, McCauley R, et al. Branched-chain amino acids. J Gastroenterol Hepatol. 2000;15:706–717. 10937674 10.1046/j.1440-1746.2000.02205.x

[4] Anthony JC, Anthony TG, Kimball SR, et al. Orally administered leucine stimulates protein synthesis in skeletal muscle of postabsorptive rats in association with increased eif4f formation. J Nutr. 2000;130:139–145. 10720160 10.1093/jn/130.2.139

[5] Matsumoto K, Koba T, Hamada K, et al. Branched-chain amino acid supplementation attenuates muscle soreness, muscle damage and inflammation during an intensive training program. J Sports Med Phys Fitness. 2009;49:424–431. 20087302

[6] Ra SG, Miyazaki T, Kojima R, et al. Effect of bcaa supplement timing on exercise-induced muscle soreness and damage: a pilot placebo-controlled double-blind study. J Sports Med Phys Fitness. 2018;58:1582–1591. 28944645 10.23736/S0022-4707.17.07638-1

[7] Fouré A, Nosaka K, Gastaldi M, et al. Effects of branched-chain amino acids supplementation on both plasma amino acids concentration and muscle energetics changes resulting from muscle damage: a randomized placebo controlled trial. Clin Nutr. 2016;35:83–94. 25886707 10.1016/j.clnu.2015.03.014

[8] VanDusseldorp TA, Escobar KA, Johnson KE, et al. Effect of branched-chain amino acid supplementation on recovery following acute eccentric exercise. Nutrients. 2018;10:1389. 10.3390/nu10101389 PMC6212987 30275356

[9] Tartibian B, Maleki BH, Abbasi A. The effects of ingestion of omega-3 fatty acids on perceived pain and external symptoms of delayed onset muscle soreness in untrained men. Clinical J Sport Med. 2009;19:115–119. 19451765 10.1097/JSM.0b013e31819b51b3

[10] Tsuchiya Y, Yanagimoto K, Nakazato K, et al. Eicosapentaenoic and docosahexaenoic acids-rich fish oil supplementation attenuates strength loss and limited joint range of motion after eccentric contractions: a randomized, double-blind, placebo-controlled, parallel-group trial. Eur J Appl Physiol. 2016;116:1179–1188. 27085996 10.1007/s00421-016-3373-3 PMC4875060

[11] Ochi E, Tsuchiya Y, Yanagimoto K. Effect of eicosapentaenoic acids-rich fish oil supplementation on motor nerve function after eccentric contractions. J Int Soc Sports Nutr. 2017;14:23. 28717347 10.1186/s12970-017-0176-9 PMC5508798

[12] Tsuchiya Y, Yanagimoto K, Ueda H, et al. Supplementation of eicosapentaenoic acid-rich fish oil attenuates muscle stiffness after eccentric contractions of human elbow flexors. J Int Soc Sports Nutr. 2019;16:19. 30987668 10.1186/s12970-019-0283-x PMC6466674

[13] Ling PR, Boyce P, Bistrian BR. Role of arachidonic acid in the regulation of the inflammatory response in tnf-alpha-treated rats. JPEN J Parenter Enteral Nutr. 1998;22:268–275. 9739028 10.1177/0148607198022005268

[14] Dunn-Lewis C, Kraemer WJ, Kupchak BR, et al. A multi-nutrient supplement reduced markers of inflammation and improved physical performance in active individuals of middle to older age: a randomized, double-blind, placebo-controlled study. Nutr J. 2011;10:90. 21899733 10.1186/1475-2891-10-90 PMC3180350

[15] Leahy DT, Pintauro SJ. Branched-chain amino acid plus glucose supplement reduces exercise-induced delayed onset muscle soreness in college-age females. ISRN Nutr. 2013;2013:921972. 24967261 10.5402/2013/921972 PMC4045268

[16] Ra SG, Miyazaki T, Ishikura K, et al. Combined effect of branched-chain amino acids and taurine supplementation on delayed onset muscle soreness and muscle damage in high-intensity eccentric exercise. J Int Soc Sports Nutr. 2013;10:51. 24195702 10.1186/1550-2783-10-51 PMC3827986

[17] Shirato M, Tsuchiya Y, Sato T, et al. Effects of combined β-hydroxy-β-methylbutyrate (hmb) and whey protein ingestion on symptoms of eccentric exercise-induced muscle damage. J Int Soc Sports Nutr. 2016;13:7. 26933398 10.1186/s12970-016-0119-x PMC4772288

[18] Rahimi MH, Shab-Bidar S, Mollahosseini M, et al. Branched-chain amino acid supplementation and exercise-induced muscle damage in exercise recovery: a meta-analysis of randomized clinical trials. Nutrition. 2017;42:30–36. 28870476 10.1016/j.nut.2017.05.005

[19] Bongiovanni T, Genovesi F, Nemmer M, et al. Nutritional interventions for reducing the signs and symptoms of exercise-induced muscle damage and accelerate recovery in athletes: current knowledge, practical application and future perspectives. Eur J Appl Physiol. 2020;120:1965–1996. 32661771 10.1007/s00421-020-04432-3

[20] Chen TC, Chen HL, Lin MJ, et al. Potent protective effect conferred by four bouts of low-intensity eccentric exercise. Med Sci Sports Exerc. 2010;42:1004–1012. 19997007 10.1249/MSS.0b013e3181c0a818

[21] Sasaki K, Sasaki T, Ishii N. Acceleration and force reveal different mechanisms of electromechanical delay. Med Sci Sports Exerc. 2011;43:1200–1206. 21200348 10.1249/MSS.0b013e318209312c

[22] Ochi E, Tsuchiya Y, Nosaka K. Differences in post-exercise t2 relaxation time changes between eccentric and concentric contractions of the elbow flexors. Eur J Appl Physiol. 2016;116:2145–2154. 27632383 10.1007/s00421-016-3462-3

[23] Howatson G, Hoad M, Goodall S, et al. Exercise-induced muscle damage is reduced in resistance-trained males by branched chain amino acids: a randomized, double-blind, placebo controlled study. J Int Soc Sports Nutr. 2012;9:20. 22569039 10.1186/1550-2783-9-20 PMC3395580

[24] Jackman SR, Witard OC, Jeukendrup AE, et al. Branched-chain amino acid ingestion can ameliorate soreness from eccentric exercise. Med Sci Sports Exerc. 2010;42:962–970. 19997002 10.1249/MSS.0b013e3181c1b798

[25] Warren GL, Lowe DA, Hayes DA, et al. Excitation failure in eccentric contraction-induced injury of mouse soleus muscle. J Physiol. 1993;468:487–499. 8254518 10.1113/jphysiol.1993.sp019783 PMC1143838

[26] Malm C. Exercise-induced muscle damage and inflammation: fact or fiction? Acta Physiol Scand. 2001;171:233–239. 11412135 10.1046/j.1365-201x.2001.00825.x

[27] Proske U, Morgan DL. Muscle damage from eccentric exercise: mechanism, mechanical signs, adaptation and clinical applications. J Physiol. 2001;537:333–345. 11731568 10.1111/j.1469-7793.2001.00333.x PMC2278966

[28] Chleboun GS, Howell JN, Conatser RR, et al. Relationship between muscle swelling and stiffness after eccentric exercise. Med Sci Sports Exerc. 1998;30:529–535. 9565934 10.1097/00005768-199804000-00010

[29] Nicastro H, da Luz CR, Chaves DF, et al. Does branched-chain amino acids supplementation modulate skeletal muscle remodeling through inflammation modulation? Possible mechanisms of action. J Nutr Metab. 2012;2012:136937. 22536489 10.1155/2012/136937 PMC3321450

[30] Tartibian B, Maleki BH, Abbasi A. Omega-3 fatty acids supplementation attenuates inflammatory markers after eccentric exercise in untrained men. Clinical J Sport Med. 2011;21:131–137. 21358504 10.1097/JSM.0b013e31820f8c2f

[31] Sharp CP, Pearson DR. Amino acid supplements and recovery from high-intensity resistance training. J Strength Cond Res. 2010;24:1125–1130. 20300014 10.1519/JSC.0b013e3181c7c655

[32] Tsuchiya Y, Ueda H, Yanagimoto K, et al. 4-week eicosapentaenoic acid-rich fish oil supplementation partially protects muscular damage following eccentric contractions. J Int Soc Sports Nutr. 2021;18:18. 33648546 10.1186/s12970-021-00411-x PMC7923476

[33] Gray P, Chappell A, Jenkinson AM, et al. Fish oil supplementation reduces markers of oxidative stress but not muscle soreness after eccentric exercise. Int J Sport Nutr Exerc Metab. 2014;24:206–214. 24225668 10.1123/ijsnem.2013-0081

[34] Nosaka K, Sacco P, Mawatari K. Effects of amino acid supplementation on muscle soreness and damage. Int J Sport Nutr Exerc Metab. 2006;16:620–635. 17342883 10.1123/ijsnem.16.6.620

[35] Nosaka K, Clarkson PM. Changes in indicators of inflammation after eccentric exercise of the elbow flexors. Med Sci Sports Exerc. 1996;28:953–961. 8871903 10.1097/00005768-199608000-00003

